# Designing Molecular Dynamics Simulations to Shift Populations of the Conformational States of Calmodulin

**DOI:** 10.1371/journal.pcbi.1003366

**Published:** 2013-12-05

**Authors:** Ayse Ozlem Aykut, Ali Rana Atilgan, Canan Atilgan

**Affiliations:** Faculty of Engineering & Natural Sciences, Sabanci University Tuzla, Istanbul, Turkey; UNC Charlotte, United States of America

## Abstract

We elucidate the mechanisms that lead to population shifts in the conformational states of calcium-loaded calmodulin (Ca^2+^-CaM). We design extensive molecular dynamics simulations to classify the effects that are responsible for adopting occupied conformations available in the ensemble of NMR structures. Electrostatic interactions amongst the different regions of the protein and with its vicinal water are herein mediated by lowering the ionic strength or the pH. Amino acid E31, which is one of the few charged residues whose ionization state is highly sensitive to pH differences in the physiological range, proves to be distinctive in its control of population shifts. E31A mutation at low ionic strength results in a distinct change from an extended to a compact Ca^2+^-CaM conformation within tens of nanoseconds, that otherwise occur on the time scales of microseconds. The kinked linker found in this particular compact form is observed in many of the target-bound forms of Ca^2+^-CaM, increasing the binding affinity. This mutation is unique in controlling C-lobe dynamics by affecting the fluctuations between the EF-hand motif helices. We also monitor the effect of the ionic strength on the conformational multiplicity of Ca^2+^-CaM. By lowering the ionic strength, the tendency of nonspecific anions in water to accumulate near the protein surface increases, especially in the vicinity of the linker. The change in the distribution of ions in the vicinal layer of water allows N- and C- lobes to span a wide variety of relative orientations that are otherwise not observed at physiological ionic strength. E31 protonation restores the conformations associated with physiological environmental conditions even at low ionic strength.

## Author Summary

Calmodulin (CaM) is involved in calcium signaling pathways in eukaryotic cells as an intracellular Ca^2+^ receptor. Exploiting pH differences in the cell, CaM performs a variety of functions by conveniently adopting different conformational states. We aim to reveal pH and ionic strength (IS) dependent shifts in the populations of conformational substates by modulating electrostatic interactions amongst the different regions of the protein and with its vicinal water. For this purpose, we design extensive molecular dynamics simulations to classify the effects that are responsible for adopting different conformations exhibited in the ensemble of NMR structures reported. Lowering the IS or pH, CaM experiences higher inter-lobe orientational flexibility caused by extreme change in the non-specific ion distribution in the vicinal solvent. Amongst the titratable groups sensitive to pH variations, E31 is unique in that its protonation has the same effect on the vicinal layer as increasing the IS. Furthermore, E31A mutation causes a large, reversible conformational change compatible with NMR ensemble structures populating the linker-kinked conformations. The mutation in the N lobe, at a significant distance, both modulates the electrostatic interactions in the central linker and alters the EF-hand helix orientations in the C lobe.

## Introduction

Protein behavior in solution may be manipulated and controlled through tailored structural perturbations [1] and rational control of the solution conditions [2] http://www.pnas.org/content/109/50/E3454.full.pdfhtml. In the living cell, proteins adapt to particular subcellular compartments which pose different environmental variables such as pH and ionic strength (IS), adapting their biophysical characteristics to tolerate pH fluctuations that are caused by cellular function [3]. Furthermore, proteins interact with many other biological macromolecules while they are transferred from one compartment to another, with subtle control over protonation and pK changes upon binding to other proteins and ligands [4], [5]. Interactions with the environment and other molecules are closely related to local anisotropy and dynamical heterogeneity of proteins [6]. The dynamics may be electrostatically guided, perhaps through long-range electrostatic interactions that select and bring interacting partners together, steering the protein to alternative conformations [7].

The main perturbation effect of the long-range electrostatic interactions is manifested on the acidic/basic groups in the protein which can be charged or neutral in relation to their conformation dependent pKa values [8]. Interacting with other molecules and changes in the environmental variables such as subcellular localization can induce shifts in ionization states of charged groups on a protein by proton uptake/release. Such changes facilitate the protein to span a large conformational space and enable it to participate in diverse interaction scenarios. Any in depth understanding developed through studying the conformational changes in proteins induced by shifts in the charge states of select amino acids would contribute to our knowledge base on diverse functionality observed in promiscuous proteins [9].

In this study, we focus on the conformation-related effects of introducing perturbations on charged group(s) of calmodulin (CaM). CaM is a notorious example among proteins having the ability to change conformation upon binding to diverse ligands [10], [11]. It was shown that negatively charged side chains in calcium loaded CaM (Ca^2+^-CaM) are attracted to positively charged residues in many of its targets [12]. Another study showed that Ca^2+^-CaM changes conformation when introduced to a solvent at low pH and low ionic strength [13]. Also, it was proposed that electrostatic interactions between acidic residues in CaM contribute to determining the most populated conformation under varying solution conditions [13].

Previously, we have studied the conformational changes in Ca^2+^-CaM [14], ferric binding protein [15] and a set of 25 proteins that display a variety of conformational motions upon ligand binding (e.g., shear, hinge, allosteric) [16] using the perturbation response scanning method. This coarse grained methodology is based on the assumption that the equilibrium fluctuations at a given local free energy minimum of the protein possess information on other viable conformations when an external force is applied [15]–[17]. Our study on CaM determined key residues that lead to the experimentally observed conformational changes upon application of force in specific directions [14]. Several different servers (H++ [18], propKa 2.0 [19], pKd [20] and PHEMTO [21], [22]) showed that the pKa of E31 value is upshifted; furthermore, the equivalent position in Calbindin was measured to have pKa of 6.5 [23]. In a follow-up study, we focused on residues with upshifted pKa values and we made a systematic study of the dynamics of Ca^2+^-CaM on time scales up to 200 ns for three separate initial configurations; extended form, compact form and extended structure with 10 protonated residues (9 acidic residues and a histidine) [24]. We found that Ca^2+^-CaM with 10 protonated residues undergoes a large conformational shift from the extended structure to a relatively compact form on the time scale of tens of nanoseconds. The latter was compatible with other structures reported in a nuclear magnetic resonance (NMR) ensemble of CaM [25].

Experimental work investigating dynamical behavior of Ca^2+^-CaM has shown that it occupies a number of hierarchical set of substates even in the crystal form [26]. Dynamical information obtained from fluorescence resonance energy transfer (FRET) experiments measuring the distance distributions between labeled sites illustrate that at least two conformations exist in solution under physiological conditions [27]. More recently, pseudo contact shifts and residual dipolar couplings of the C-terminal domain obtained using NMR [28] revealed neither the dumbbell shaped conformation observed in early crystal structures of the molecule [29], nor the compact conformation determined later on [30] exist in significant proportions in solution. Ca^2+^-CaM is identified as a protein which populates multiple conformations [28], [31]. A shift between the distribution of populations is induced by changing environmental conditions such as pH, Ca^2+^ concentrations and ionic strength [13], [27]. Each of these manipulated properties has effects on the charged groups of Ca^2+^-CaM.

The presence of multiple conformations is a physical property of Ca^2+^-CaM, and it is likely that the heterogeneity of structure is at least partially responsible for the ability of Ca^2+^-CaM to recognize diverse targets. Squier and coworkers have suggested that association of the C-terminal domain of CaM with a target may disrupt a structurally important hydrogen bond involving the central linker, facilitating formation of a compact binding conformation of Ca^2+^-CaM [32]. More recently, through rather benign mutations such as E47D, they have determined noninterfacial residues important for molecular recognition through indirect effects – an increase in fluctuations stabilizes the bound state [33]. It was further hypothesized that pH and ionic strength dependent shifts in the populations of conformational substates result from changes in electrostatic interactions in the central linker [13], [27]. For example, the shift in favor of the more compact conformation at reduced pH may result from the loss of electrostatic interactions that serve as spacers at neutral pH. This hypothesis is corroborated by inspection of the proximity of side chains of glutamic and aspartic acid residues surrounding the hinge region in the compact Ca^2+^-CaM crystal structure [30].

In this manuscript, we report extensive molecular dynamics (MD) simulations of fully solvated, extended and compact Ca^2+^-CaM under different perturbation scenarios, with focus on E31. We have previously shown that E31 is located in a unique position to manipulate the overall structure; it also has an upshifted pKa into the physiological range and there are several experiments implicating its involvement in signaling coordination between the two lobes (see [14] and references cited therein). Structural perturbations are introduced as either E31A mutation or its protonation. We also perturb environmental factors such as pH and IS. We analyze the structural dynamics through identifiers based on reduced degrees of freedom defined specifically for Ca^2+^-CaM. Key events leading to or preventing conformational change are discussed. We elaborate on the events occurring along the path sampled between different conformational states identified by MD simulations and we evaluate the effect of charge balance on the conformations. The molecular mechanisms that lead to the observed effects, their relationship to the experimental data, and the consequences of the observations that enhance our understanding of the dynamics and function of Ca^2+^- CaM are outlined.

## Methods

### CaM structures and definition of low resolution degrees of freedom

CaM consists of 148 amino acids made up of the N-lobe (residues 1–68), the C-lobe (residues 92 to 148) and a linker which is helical in many, but not all, of the reported structures. Each lobe in CaM has two helix-loop-helix (EF-hand motif) calcium binding sites connected by unstructured sequences. Structured elements include helices I (residues 5–17), II (residues 30–39), III (residues 46–54), IV (residues 69–73), V (residues 83–91), VI (residues 101–110), VII (residues 119–129), and VIII (residues 137–144). Ca^2+^ coordinating residues in each of the four EF-hands are D20-D22-D24-E31 in loop I, D56-D58-N60-E67 in loop II, D93-D95-N97-E104 in loop III, and D129-D131-D133-E140 in loop IV. All MD simulations reported in this work include the four Ca^2+^ ions.

The existing X-ray structures of Ca^2+^-loaded, peptide free calmodulin (Ca^2+^-CaM) are either in an extended or a compact form. There are many examples for the extended form in the protein data bank (PDB) and we utilize that with PDB code 3CLN whereby the coordinates of the first four and the last residue are not reported [29]. The compact form is represented by the 1PRW coded structure [30], and has a bent linker as do many ligand bound Ca^+2^-CaM conformations present in the PDB. These particular structures have been determined at 2.2 and 1.7 Å resolution, respectively, and were both crystallized at low pH conditions in the range of 5–6, by growth in water-organic mixture compounds. We have previously reported the RMSD comparison for the overall structure as well as the N- and C-lobes of various x-ray structures, including 3CLN, 1PRW and five ligand bound forms [14].

An ensemble of Ca^2+^-CaM structures have also been reported (PDB code 2K0E) [25] by using experimental NMR order parameters (*S* ^2^) together with interproton distances derived from nuclear Overhauser effects (NOEs) as restraints in MD simulations using RDC-refined solution structure of Ca^2+^-CaM. The IS of the experimental setup is 10 mM and the pH is 7 (the conditions in ref. [25] are the same as in ref. [34]; personal communication). The ensemble has 160 structures and reveals that Ca^2+^-CaM state samples multitude of conformations including, but not limited to, the compact and extended ones. In particular, unlike in the X-ray structures, there also exist compact conformers where the linker is not bent, as we pointed out in our previous study [24].

**Figure 1:**
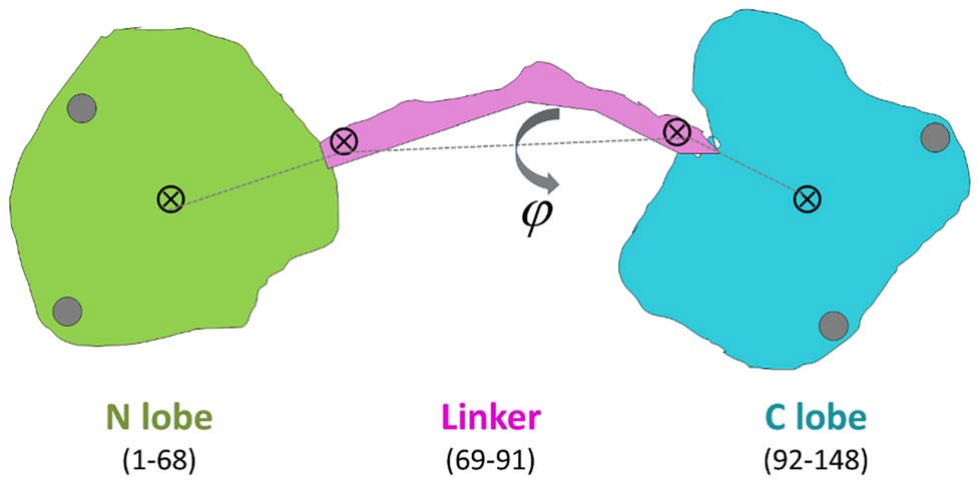
Schematic display of Ca^+2^-CaM displaying the color code used throughout the text (green for N lobe, purple for linker, cyan for C-lobe; gray circles represent Ca^+2^ ions), the four points used to define the reduced degrees of freedom (shown by ⊗) are the centers of mass of the N- and C-lobes and the beginning and end points of the linker. We use the linker length *l*, measured by the distance between the latter two, and the linker torsion angle *φ*, defined on the figure, as the major degrees of freedom where we project all trajectories to monitor the sampled conformations.

Throughout this work, an efficient approach to distinguish between the different conformations of CaM proves to be useful: We define two low resolution degrees of freedom projecting the 3*N*-dimensional conformational space into a visually tractable two-dimensional one. These are the linker end-to-end distance (*l*) and torsion angle (*φ*). The former is defined as the distance between the C_α_ atoms of the two outermost residues of the linker, residues 69 and 91. The latter is the torsion angle defined by four points: the center of mass of the N-lobe (residues 5 to 68), linker beginning and end points (C_α_ atoms of residues 69 and 91), the center of mass of the C-lobe (residues 92 to 147). These points are schematically shown in Figure 1.

### MD simulation details

Six sets of simulations were performed with various initial starting conditions. Simulations are summarized in Table 1; IS values reported correspond to the equilibrated box dimensions. For some systems, we have performed independent runs to check the reproducibility of the results. Each condition has at least 150 ns of total sampling time. We prolong the simulation in case there are substantial changes in the relative positioning of the two lobes and/or the length of the linker, measured by the region sampled on the (*l*, *φ*) plane described in the previous subsection.

**Table 1:** Summary of system parameters and perturbation scenarios.

Starting Conformation	Perturbation Scenario^a^	Ionic strength (mM)	Number of atoms	Equilibrated Box size (Å)	Simulation length (ns)	System label^b^
Extended (3CLN)	–	150	33835	82×84×90	150	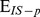
					50	
Compact (1PRW)	–	161	20330	58×57×60	200	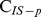
					200	
Extended (3CLN)	–	82	37099	62×87×69	400	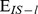
					50	
Extended (3CLN)	11, 31, 67, 84, 93, 104, 107, 122, 133, 140 are protonated	43	31660	64×81×60	200	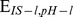
					100	
Extended (3CLN)	E31 is protonated	91	31652	63×81×60	400	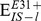
					150	
Extended (3CLN)	E31A mutation	94	31645	60×83×62	400	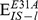
					400	
					400	

= 7.4 using pK_a_ values calculated in ref 22. These systems are assumed to be at physiological pH.^a^ Unless otherwise indicated, residues are assigned charge states according to pH

*IS-p*: physiological ionic strength, *IS-l*: low ionic strength, *E*31+: residue E31 is protonated, *E*31*A*: residue E31 is mutated to A, pH – *l*: low pH.^b^ Abbreviations: E: Extended, C: Compact;

The details of each simulation are as follows; the label for each type of simulation is indicated in parentheses and will be used throughout the text:

(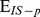) Initial coordinates are taken from the extended 3CLN pdb coded structure and all residues are assigned their standard protonation states to study the conformational dynamics of extended form in solution. 45 Na^+^ and 30 Cl^-^ ions are added to attain IS = 150 mM at the physiological range. There is a one MD run of 150 ns and an additional control run of 50 ns.

(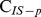) Initial coordinates are taken from the compact 1PRW and all residues are assigned their physiological protonation states to study the dynamics of compact structure in solution. The system is neutralized by 15 Na^+^ ions. Due to the smaller box dimensions formed for this more compact structure, this protocol leads to IS = 161 mM at the physiological range. There are two runs of 200 ns each for this system.

(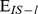) Starting from 3CLN and all residues having the same protonation states as in (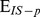), the system is neutralized by 15 Na^+^ ions. This leads to a low IS of 82 mM. There is a on MD run of 400 ns and an additional control run of 50 ns.

(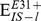) Starting from 3CLN structure, only E31 is protonated. The system is neutralized by 14 Na^+^ ions leading to IS = 91 mM; there are two runs for this system, one of length 400 ns and the other of 150 ns.

(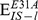) Starting from 3CLN structure, E31A mutation is made. The system is neutralized by 14 Na^+^ ions leading to IS = 94 mM; there are three runs of 400 ns each for this system.

(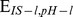) In all the previously listed simulations, residues are assigned charge states according to pH = 7.4 using pK_a_ values calculated and listed in ref. [24]. These systems are assumed to be at physiological pH. Acidic residues 11, 31, 67, 84, 93, 104, 122, 133 and 140, as well as H109 consistently have pKas shifted from their standard values to ∼5.5. In the 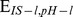 system, these are protonated to mimic the low pH conditions. The reader is referred to ref. [24] for details on the calculation of pKa values. There are two runs for this system, one of length 200 ns and the other of 100 ns. The system is neutralized by 5 Na^+^ ions leading to IS = 43 mM

In addition, control runs of 100 ns duration have been carried out on the extended, low IS proteins, singly or doubly protonating other residues with upshifted pKa values. These are labeled 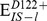, 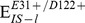, 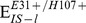 and are not separately listed in Table 1; they are neutralized by 14, 13 and 13 Na^+^ ions, respectively.

We use the NAMD package to model the dynamics of the protein-water systems [35]. The protein is soaked in a water box with at least 10 Å of water from all directions using VMD 1.8.7 program with solvate plug-in version 1.2 [36]. The CharmM22 force field parameters are used for the protein and water molecules [37]. Water molecules are described by the TIP3P model. Each system is neutralized by using VMD autoionize plug-in. Long-range electrostatic interactions are calculated by the particle mesh Ewald sum method, with a cutoff distance of 12 Å and a switching function of 10 Å [38]. RATTLE algorithm is utilized and a step size of 2 fs is used in the Verlet algorithm [39]. Temperature control is carried out by Langevin dynamics with a damping coefficient of 5/ps. Pressure control is attained by a Langevin piston. Volumetric fluctuations are preset to be isotropic. The system is run in the NPT ensemble at 1 atm and 310 K. Equilibration of the pressure is achieved within 2 ns. The equilibrated box dimensions of each system are listed in Table 1. The coordinate sets are saved at 2 ps intervals for further analysis.

## Results

We seek the conditions under which the conformations sampled by Ca^+2^-CaM may be manipulated under different perturbation scenarios. In what follows, we show that a starting conformation may be destabilized by changing the pH of the environment as well as reducing the IS. Moreover, large conformational changes may be delimited or induced by targeting perturbation-sensitive residues selected based-on a coarse-grained analysis of relatively short trajectories.

**Figure 2:**
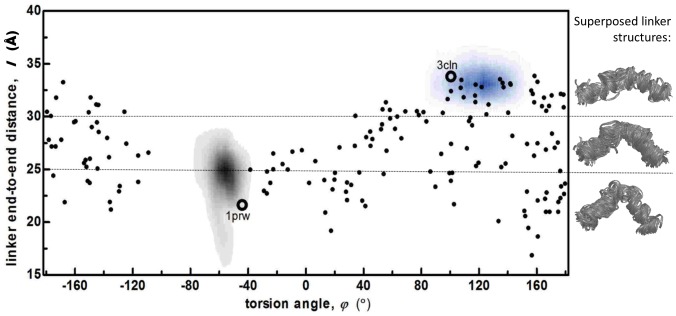
Conformations sampled by extended and compact Ca^+2^-CaM at physiological IS (labels 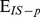 and 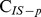, shown by gray and blue shades, respectively) displayed on torsional angle *φ* vs. linker end-to-end distance *l* plots. Also marked are the wild CaM extended (3CLN), compact (1PRW) crystal structures (black empty circles) and the 160 structures from the 2K0E NMR ensemble (black dots). Shown on the right are the superposed linker structures of the 2K0E NMR ensemble, grouped according to linker end-to-end distance *l*; number of structures with *l*>30 Å, 25<*l*<30Å, and *l*<25 Å are 48, 57 and 55, respectively. For linker lengths less than 30 Å, the bending always occurs at residues 79–81.

We display in figure 2, the locations of experimental structures on the reduced conformational space for 3CLN, 1PRW (the extended and compact crystal structures, respectively) and 2K0E (160 NMR solution structures). There are few conformational representatives in solution that are close to the X-ray structures. Also displayed on the right of the figure is the superposition of the linker conformations of the 2K0E ensemble. We find that for linker lengths less than 30 Å, the bending is always in the same direction, and occurs at residues 79–81, although there is no local salt bridge stabilizing it.

Overlaid on these points are the regions sampled by the 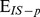 and 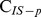 MD simulations clearly showing that at physiological pH and IS, the Ca^2+^-CaM conformations are confined near their initial states in all four runs at 150 mM IS. Thus, these structures are deemed stable around the initial conformations under physiological conditions on the time scales of 100 ns. This is an expected outcome, since conformational jumps between states were measured to occur on the 100 µs time scale from single molecule experiments at physiological pH and IS [27]. Note that the sampled regions overlap with only fourteen of the 160 NMR solution structures that are compatible with experimental measurements for the extended and only one for the compact conformer.

### Redistribution of conformational states and time scales by modifying environmental conditions

Starting from 3CLN which represents the extended Ca^+2^-CaM structure captured in most X-ray studies, we externally perturb the physiological conditions for which results were displayed in figure 2: (i) We lower the IS while keeping the pH at 7.4; and (ii) we lower the IS as well as reducing the pH to 5.0 [24]. These systems are labeled 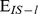 and 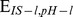, respectively (Table 1).

**Figure 3:**
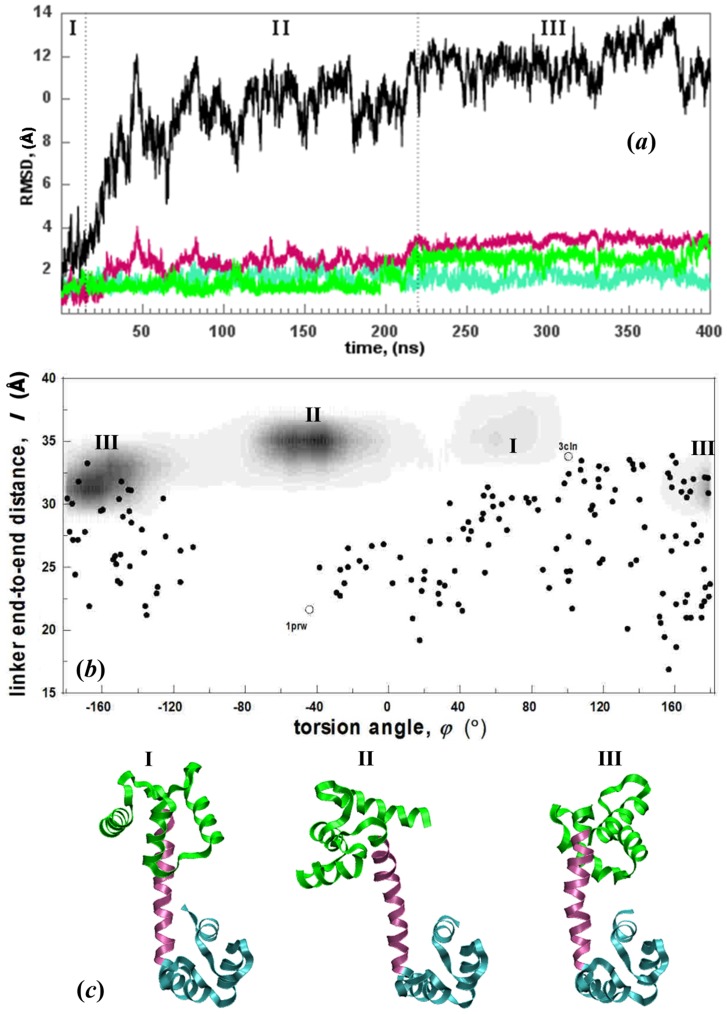
Conformations sampled by 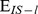
system. (a) RMSD of subunits depicted schematically in figure 1 (same color code) as well as the total structure (black) reveals that the intradomain motions are small, while their relative orientations change substantially during the 400 ns trajectory. The three major conformations sampled are depicted as regions I, II and III. (b) (*φ*,*l*) plots of 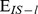 (gray shaded areas) overlayed on experimental structures as in Figure 2; regions I, II and III depicted in (a) are easily distinguished. (c) Structures exemplifying regions I, II and III.

The regions sampled by 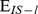 are displayed in figure 3. This is a continuation of the MD simulations from our previous work [24], where the run has now been extended from 200 ns to 400 ns. The RMSD values of the subunits as well as the overall structure are shown in figure 3a. The initial conformer is not stable in solution as confirmed by the protein RMSD change. While the linker and the two lobes each display low intra-domain motions (less than 3±1 Å RMSD), their relative orientations change substantially (up to 13 Å in the overall RMSD). When projected on the reduced degrees of freedom (figure 3b), the trajectories clearly display the three separate sampled states: The two lobes initially point towards each other and within the first 25 ns, the N- and C-lobes complete a ca. 120° torsional motion reaching state II which is then sampled for 195 ns after which a new state is reached (III) by a further torsional motion of 100°. In the last 180 ns of the trajectory, state III is sampled. Snapshots exemplifying these three distinct states are shown in figure 3c. We note that a prompt move into region II also occurs in the supplementary 50 ns run.

All the structures that are sampled throughout the MD trajectory are compared with the experimental ones. The protein spends the first 220 ns in intermediate states with linker length (*l*>31 Å), and *φ* = [−80°, 100°]. During the MD simulation, the initial (X-ray derived) structure 3CLN is only transient and the conformations sampled in regions I and II do not overlap with any of those from the NMR ensemble. This is consistent with other NMR and single molecule experiments where very low occupancy is assigned to the fully extended structure [31]. After 220 ns, the system eventually relaxes into a region with *l* = [28], [34] Å, *φ* = [−210°, −130°] which overlaps with many of the 2K0E NMR ensemble members (figure 3b). These observations imply that there is an energy barrier between the regions with *φ* = 90° and *φ* = 150° so that the system must counter-rotate by a large torsional angle, instead of flipping the 60° directly.

In the 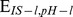 runs, we find that by mimicking low pH, low IS environment, the sampled regions in figure 3b do not change (see figure S1), but the sampling is accelerated. We do not go into the details of these runs since we have already published a detailed account of the conformations sampled and key events leading to the conformational change [24]. However, it suffices to say that the same sequence of states I→II→III are followed in both 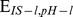 runs. The shift from state I to II occurs at ca. 20 ns similar to the time scales observed in 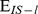, but that from II to III occurs at ca. 70 ns.

For both the 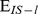 and the 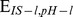 systems, the main intra-domain conformational change occurs as a reorientation of helix II in the N-lobe. For example, the nearly right angle between helices I-II, that was always maintained at nearly right angles (80±6°) at physiological IS in the 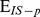 and 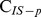 runs, is now reduced within the first 10–20 ns of the trajectory. It is maintained at a value of 56±7° in 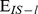 and 60±5° in 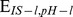 throughout the window of observation. The major event that stabilizes the closed conformation is the formation of salt bridge(s) between the N-lobe and the linker in each case: E7-K77, E11-K77 or E54-K75 in 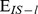 (established in both runs prior to 50 ns) and E7-R74 or E11-K77 in the 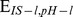 runs (forming permanently at ca. 40 ns in both samples).

### Conformational motions are diversified by altered charge distribution on the surface

Despite being a counterintuitive observation, it was shown as a general result that two negatively charged nano-sized spheres may be put into close contact by utilizing the competition of hydrophobic and Coulombic interactions, provided that the charges are placed discretely along the surface [40]. At physiological IS and pH, there exists a high energy barrier between the extended and compact structures, corroborated by the 100 µs time scale of jumps between them, measured by single molecule experiments [27]. Thus, a direct passage between the black and blue shaded regions in figure 2 is not observed within the time window of observations of the MD simulations. However, once achieved, the compact conformation is stable despite the net repulsions between the two lobes (net charge on the N- and the C-lobes are -8 and -6, respectively).

One may argue that the pH of the X-ray experiment (5.4) may have contributed to the stability of the 1PRW crystal structure, since the acidic residues 7, 11, 14, 120, 127 are found to be neutral at this pH [41]. The interface between the two lobes involves E7 and E11 on the N-lobe interacting with E127 on the C-lobe, as well as pairing between E14-E120. Thus, it may well be that the acidic contacts do not repel each other in the crystal due to the loss of the charges. In contrast, our MD simulations starting from the 1PRW structure assigns their usual charges to these residues to mimic the physiological pH conditions. Nevertheless, the interface accommodates the repulsions between the closely located negative charges by slightly expanding around the adjacent helices and rotations in the side chains (figure S2). Thus, the initial state is maintained during the 200 ns window of MD observations, regardless of the charge states of the interface residues.

With this robust accommodation of charges in mind, we seek the reasons behind the relaxation of the initial X-ray structure to new conformations when the environmental conditions are perturbed. To be noted is the conformational plasticity in the MD runs at low IS (figure 3b), and the similarity between a subset of the NMR ensemble structures and state III structures. The ionized states of the acidic residues make the electrostatic component dominant and strongly oppose direct inter-domain association on the time scale of the simulations [41]. This fact does not keep the system from sampling a plethora of conformations in the *φ* space. Thus, to understand how the interfered charge distribution in the environment affects the vicinal solvent layer around the protein, we study the distribution of the solvated non-specific ions around the protein in each case.

**Figure 4:**
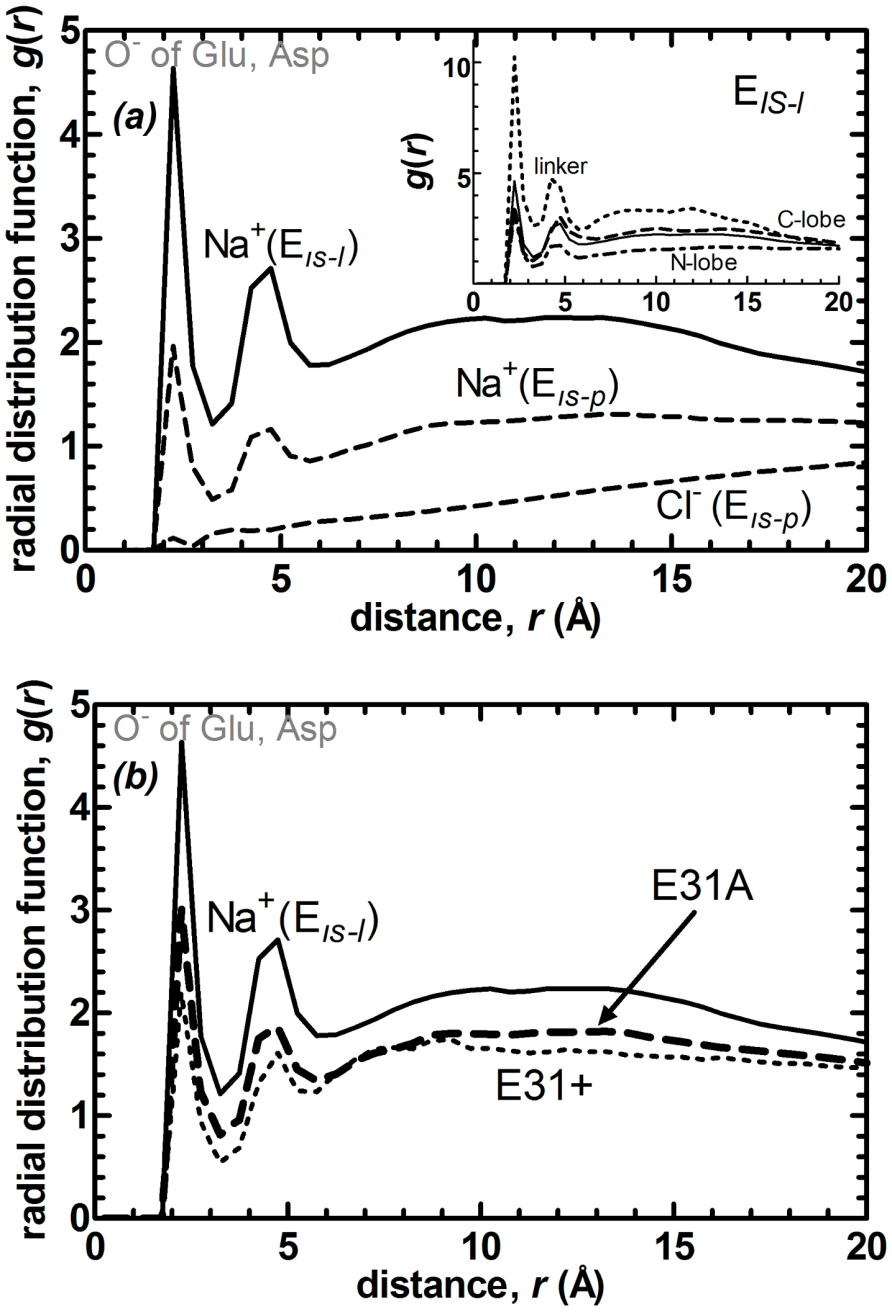
Radial distribution functions (RDFs, *g(r)*) between side-chain atoms and nonspecific ions in the solvent comparing (a) the systems at physiological and low IS, 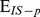 (dashed) and 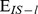 (solid line). The latter have no Cl^−^ ions in the simulations. Decreasing IS has the direct effect of accumulation of Na^+^ ions near the protein surface. Inset shows for the 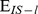 system, RDFs belonging to the linker (dotted), N-lobe (dot-dashed), C-lobe (dashed) and protein (solid line). Most of the charge accumulation occurs around the linker. (b) The systems at low IS with standard E31 (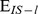, same as in part a; solid line), E31A protonation (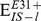, dotted), and E31A mutation (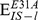). Protonation has a similar effect to increasing the IS, significantly reducing the cation accumulation around the protein. Locations of the first peaks for these and other systems are listed in Table 2.

We display in figure 4a, the radial distribution function (RDF) of the ions in the solvent around the side chain heavy atoms of the protein at low and high IS. The first peak belongs solely to the contact of Na^+^ ions with O^−^ atoms of the negatively charged residues. The second peak is due to the solvent mediated interactions. Interestingly, although there are plenty of positively charged residues on the surface of the protein, Cl^−^ ions (which only exist in the physiological IS run) rarely interact with them. At low IS, the Na^+^ ions strongly interact with the negative charges on the protein, thus screening the extreme repulsions between the two lobes and allowing rotational motions around the linker. To achieve physiological IS, Cl- ions as well as additional Na+ ions are added to the system. In the presence of these additional mobile negative charges the Na^+^ ions mainly reside away from the protein surface and in bulk water where they may also dynamically interact with Cl^−^ ions (we check that there is no permanent ion pairing occurring between Na^+^ and Cl^−^ ions). More interestingly, lack of salt in the solvent environment reduces the time scale of conformational change by three orders of magnitude, from sub-milliseconds to sub-microseconds.

Decomposed into the different regions on the protein at low IS (Figure 4a inset), the most significant interaction of the Na^+^ ions is with the linker residues, followed by those of the C-lobe and even less so with the N-lobe. We have also monitored the trajectories to find that the cations are mobile and they do not have a preferred position near the linker. These ionic distributions are contrary to expectations from the net charges, with that of the linker being only -1, whereas those of the N- and the C-lobes are -8 and -6, respectively. Thus, the ionic interactions are geometry specific, and designate the smooth surface of the linker (relative to the two lobes) as a region that has a tendency of binding non-specific ions. We conclude that the conformational plasticity of the torsional motions observed in altered charge environments is due to the clustering of the cations around the linker which screens the strong repulsions between the two lobes.

We check the effect of the change in the number densities of the ions at different ionic strengths on the values of the RDF peaks. We confirm that the reduction of the peaks exceeds that expected by the 2.3 fold increase in the number densities of the ions in the system (e.g. the linker peak is reduced 3.3 fold.) In terms of the absolute values, the average number of Na^+^ ions within the first coordination shell of the acidic residues of the linker is 0.5 and 1.13 for the 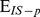 and 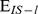 systems, respectively.

### E31 is located in a key position to disrupt the electrostatics of the whole protein

While the removal of Ca^2+^ ion from EF-hand loop I readily induced compaction of CaM in a previous MD simulation [42], we are interested in revealing its role in CaM dynamics in fully loaded state. We have previously shown that E31located in this loop is unique in that its perturbation in a given direction reproduces the closed form structure with high overlap [14]. In fact, unlike its positional counterparts on the other EF-hand loops of CaM, the role of E31 is not as central in Ca^+2^ ion coordination as its involvement in signaling coordination between the two lobes. This statement is supported by a series of experimental E→K point mutation studies at the four equivalent EF-hand positions (31, 67, 104 and 140) [43], [44]. Two results are striking: E31K mutation (i) has wild type activation on four different enzymes while the others do not; (ii) does not lead to apparent binding affinity changes while the rest lead to the loss of Ca^+2^ binding at one site. It was also shown that proton flux is an important factor affecting conformational changes in CaM and its enzyme targets [45]. We have therefore protonated E31 while keeping the IS low in the 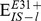 set of MD simulations.

Since the topology of the residue is the same except for the reduced charge on the side chain, it still interacts with the Ca^2+^ in the EF hand I. We have monitored this motif throughout the trajectories and ensured there is no loosening in the motif. Strikingly, we find that the net effect of this single point protonation on the sampled conformations is similar to increasing the IS, keeping them near the initial extended structure (compare figures 2b and S3b), with an average *l* value of 33 Å and torsional angle range *φ* = [80°,130°]. Despite the protonation of a single point, the RDFs measured in these runs are also more similar to the high IS system (figure 4b), significantly reducing the density of Na^+^ ion clustering around the protein, mainly affecting the linker region (value reduced to 3.1 from 10.3; see Table 2).

**Table 2:** Value of the first peak in the RDFs*.

System label	E*_IS-p_*	E*_IS-l_*	E*_IS-l,pH-l_*	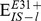	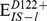	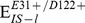	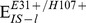	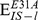
**protein**	2.0	4.6	3.6	3.5	3.2	2.9	3.2	2.8
**linker**	3.1	10.3	3.1	6.8	6.9	4.5	7.0	5.3

Figure 4. Values are confirmed by alternative runs where available or by creating at least two 50 ns long pieces out of single trajectories. Defined in

E31 is able to significantly reduce the ion density around the protein and the linker at the same time (Table 2). For example, protonation of D122, the second residue with the most significantly upshifted pKa, in a control run also leads to similar values. E31/D122 double protonation further reduces the ion density around the whole protein and the linker; while the E31/H107 double protonation does not bring in this additional effect. However, protonation of 10 residues to mimic the pH 5 environment in 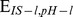 is effective in further reducing the charges around the linker environment, while its effect on the overall protein is less apparent.

### E31A mutation results in a reversible conformational change between extended and compact structure

The most drastic change in the extended conformation occurs in the 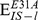 system. As we discuss in detail below, the E31A mutation opens a direct path between the extended conformation and compact structures with a bent linker, accessing conformations not sampled by any of the other systems.


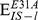
 runs are characterized by increased mobility of the N-lobe (4 Å RMSD) accompanied by an additional stability in the C-lobe (RMSD<2 Å) as well as the linker. The stability in the latter two regions, not directly perturbed by the E31A mutation, contrasts the 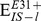 simulations discussed in the previous subsection. We emphasize that the Ca^+2^ ion coordination is never lost in any part of these MD simulations which total 0.55 µs and 1.2 µs in 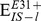 and 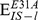, respectively. By inspecting the MD trajectories, we find that the main direct difference between 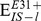 and 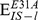 runs is that while the calcium binding motif is not disrupted in the former, residue 31 can no longer participate in the motif in the latter due to its short side chain and hydrophobic character. Interestingly, the E31A mutation restores some of the depleted charge distribution around the acidic residues that occurred upon its protonation (Figure 4b and Table 2).

**Figure 5:**
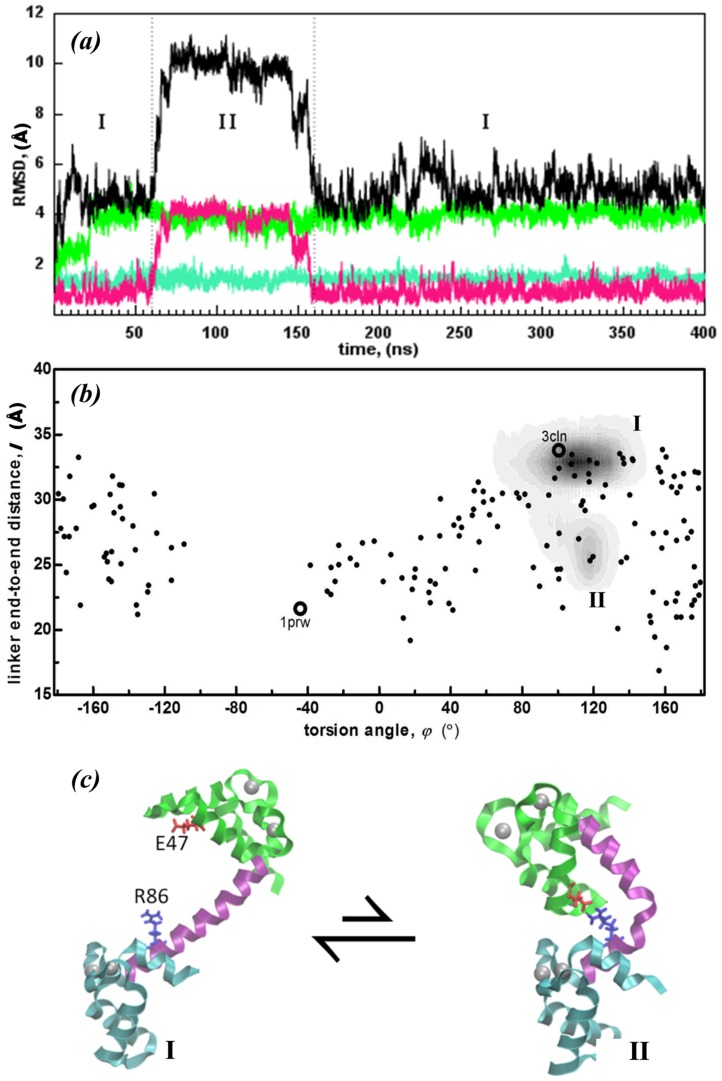
Conformations sampled by 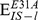 system. (a) RMSD of subunits depicted schematically in figure 1 (same color code) as well as the total structure (black) reveals that the relative orientations of the domains change substantially during the 400 ns trajectory. The two major conformations sampled are depicted by regions I, and II. (b) (*φ*,*l*) plots of 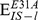 (gray shaded areas) overlaid on experimental structures as in Figure 2; regions I and II depicted in (a) are easily distinguished. (c) Sample structures denoting the two regions in this run.

The reorientation that takes place in the N-lobe is quantified by an increase of the RMSD value from 2 Å to 4 Å (Figure 5a). The angle between helices III–IV displays a drastic change, with helix III tilting towards helix IV. This is followed by the formation of a salt bridge between residues E47 and R86 at 60 ns which may be traced in the sharp decrease in *l* from 34 Å to 27 Å (Figure 5b). After the salt bridge formation, at 80 ns, the linker is further bent from residue 81 and *l* drops to 25 Å bearing a compact conformation. Snapshots are taken before and after transition and shown in Figure 5c. The observed conformational change is reversible, and the extended structure is restored at ca. 160 ns. No significant pKa shift appears for charged residues in any part of the trajectory.

**Figure 6:**
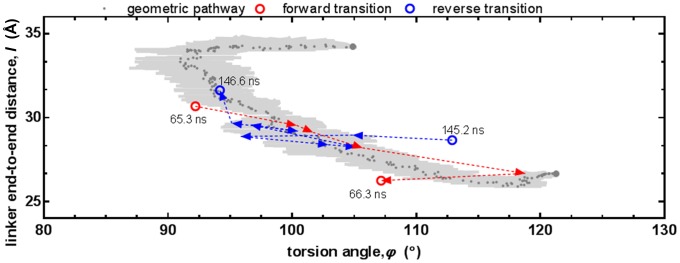
Conformational transition of the 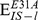 system. The transition pathway generated by the Geometrical Pathways in Biomolecules server [46] is shown in reduced degrees of freedom. The dark dray points are the median of the 10 separate runs and the light gray shaded area is the standard error bars along each axis. The initial (50 ns) and target (80 ns) structures that are fed to the server are shown with larger gray points. MD structures at several points of the trajectory are also overlayed. Forward transition (65.3–66.3 ns) and reverse transition (145.2–146.6 ns) paths shown in red and blue arrows, respectively.

We find that the transition state is well defined, occurring through the same point in both forward and reverse steps. Time intervals of the transitions between the extended and compact (forward transition) and between compact and extended conformations (reverse transition) are examined in more detail in figure 6. The positions of structures near the transition state in 200 ps intervals are plotted on the (*l*, *φ*) plane. Note that the axes have been zoomed in.

The transition between the extended and compact states is also examined via the tool Geometrical Pathways [46], [47]. This tool utilizes geometric targeting (GT) method that has recently been introduced [46] as a rapid way to generate all-atom pathways from one protein structure to some known target structure. GT is based on the philosophy that essential features of protein conformational changes can be captured by solely considering geometric relationships between atoms. The protein is modeled as a geometric system, with constraints established to enforce various aspects of structure quality such as preserving covalent bond geometry, preventing overlap of atoms, avoiding forbidden Ramachandran regions for backbone dihedral angles, avoiding eclipsed side-chain torsional angles, and maintaining hydrogen bonds and hydrophobic contacts. We note GT cannot predict relative timing of events.

Using Geometrical Pathways in Biomolecules server [46], we have generated 10 random pathways between representative structures collected structures at 50 ns (extended) and at 80 ns (compact) of the 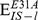 run 1. The RMSD step size is 0.05 Å. The structures generated in the forward pathway by Geometrical Pathways are also plotted on figure 6 with the median of the pathway and the standard error bars along both axes. The random pathways produced via Geometrical Pathways overlaps with that visited by MD. They are widely distributed along the interdomain torsional angle dimension, but have narrow distribution in end-to-end-linker distance. GT generated pathways take energetics into account indirectly, through geometric factors only. Their overlap with the MD pathway corroborates that the conformational change may be achieved as a series of geometrically viable sequential steps, if the energy barrier between the two states allows them to take place.

In fact, a stabilized conformational change between states I and II is observed in only one of the three 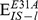 runs. However, several attempted jumps occur with a kinked linker conformation in all simulations (*l*<30 Å). Thus, the crucial step stabilizing the bent conformation is not the bending that is facilitated after the mutation, but the formation of the salt bridge between the N-lobe and the linker. Such attempts occur neither in 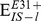 nor in physiological IS runs.

The relative positioning of the entering and exiting helices of the EF-hand motifs have been used to characterize the diverse conformations utilized by CaM for target recognition [48]. We therefore focus on the dynamics of the angles between the helices in EF hand motifs to understand how this “rare” conformational change is facilitated by E31A mutation. On the N-lobe, helices I and II in EF hand I, and helices III and IV in EF hand II are both initially posed at about right angles to each other, having values between 80–90°. In all three 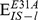 trajectories, the increase in the RMSD of the N-lobe occurs simultaneously with the loss of this perpendicular arrangement between the former pair of helices. The change in the orientation of the N-lobe helices provides E47 to make the salt bridge with R86. Perhaps most intriguing is the diminished fluctuations between helices V and VII upon E31A mutation. The values of 38±11° in the rest of the simulations of Table 1 are suppressed to 30±5° for all three 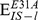.

The model for inter-lobe communication of CaM is E31 mediated. E31A mutation induces conformational changes between the two EF-hand motifs in the N-lobe, which simultaneously stabilize helix reorientational fluctuations in the C-lobe. No apparent communication pathway between the two lobes is found, thus lending support to the ensemble view of allostery [49]. As a result, the N-lobe performs an intra-lobe conformational search to establish a salt bridge with the linker. The stabilized C-lobe helps maintain the initial contact. The solvent participates in the fluctuations that establish the background for the conformations sampled by the protein.

## Discussion

Multiple conformations and cooperative conformational changes are an essential part of many enzyme mechanisms [50]. We explore the role of electrostatics in altering the conformation distributions as well as the dynamics of Ca^2+^-CaM using extensive MD simulations under physiological and low IS/pH conditions, and by mutating/protonating single residues. While the protein is stable in the initial state at physiological IS, lowering the IS or pH leads to conformational switching to more compact structures on sub-100 ns time scales (Figures 3 and S1).

Although the net charges on the N- and C-lobes are significantly higher, at low IS cations approach the linker due to its relatively smooth geometry [51], screening the repulsions between the lobes. This leads to conformational plasticity, enabling large torsional motions around the linker, eventually causing a compact conformation within 200 ns, albeit with a stiff linker. Lowering the pH in addition to the IS, which in effect deletes 10 charges on the protein surface, contributes to the process further, letting the protein achieve similar conformations within 60 ns.

At high IS, the Cl^−^ counterions are repelled by the significantly negatively charged protein (figure 4a). Na^+^ ions are then driven into the bulk solvent, since the counterion interactions are favored over those with acidic residues both energetically and entropically. Repulsion between the two lobes is more pronounced because of ion depletion in the intervening region, restricting the protein conformation near the initial structure (figure 2). It would be interesting to make a systematic study of the effect of ions on the dynamics of CaM, including different ion types and concentrations. However, our scope here is to merely demonstrate that CaM dynamics is sensitive to ionic strength and may be moderated between rigid and very flexible. Such an observation has implications on both tuning and interpreting experimental results and on conditions selected for computer simulations.

Perhaps more interestingly, neutralizing the single residue E31 at a key location by protonation has a similar effect on the ion distributions to increasing the IS (figure 4b), confining the sampled conformations near the initial structure (SI figure S3). Alanine mutation of the same residue results in an intermediate distribution of ions, leading to partial mobilization of the protein. This facilitates a bending in the linker near the extended conformation (figure 5). E31A mutation accentuates the existing allosteric interactions, by introducing a change originating on the N-lobe whose action is detected on the C-lobe via the rearrangements of the helices in the EF-hands. The coupling between the two lobes is detected simultaneously, and the stabilizing salt bridge between the N-lobe and the linker is established later on in the simulation, right before the transition state is reached (figures 5c and 6). Thus, no pathway of structural distortions between the allosteric sites is observed, lending support to the ensemble view of allostery [49].

Finally, we note that the sampled structures have representatives in the NMR ensemble of conformations [25]. 40% of the 160 conformers have been visited in the cumulative MD simulations. On the downside, these are predominantly the ones with the straight linker, while the compact conformers (those with *l*<25 Å) have been rarely observed. The structures are compatible with the FRET results both in terms of the interlobe distances and the fact that lowering the pH to 5.0 leads to a single stable state as opposed to the presence of at least two distinct forms at pH 7.4 [13]. Our observations are also compatible with the findings of Bertini and coworkers [31]. Therein, low occupancies are assigned to fully compact structure of Ca^2+^-CaM (1PRW). Extended conformers similar to 3CLN in general have low occupancies although some other extended conformers have occupancies as high as 35%. This supports our findings that in our cumulative MD trajectories, the initial structures whether starting from compact or extended crystalline structures of Ca^2+^-CaM relaxes to less extended or less compact forms.

Our current view suggests that at physiological IS and pH, there exists a barrier between the extended and compact forms, leading to 100 µs time scales for conformational jumps [27]. Barrier crossing is prevented by the repulsive electrostatic interactions between the two negatively charged lobes. As we show in this work, the crossing may be facilitated by tuning environmental conditions or by perturbing single residues located at key positions. The current study contributes to the knowledge-base in the direction of methods that determine how proteins adapt to changes in their environment or structure.

## Supporting Information

Figure S1(*φ*,*l*) plots for the two runs mimicking the low IS/low pH environment, 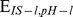. (a) 200 ns run; (b) 100 ns run. Same sequence of events is observed in these systems. Their details are discussed in [24].(TIF)

Figure S2 N- and C- lobe interface shown for the 1PRW crystal structure (transparent) [30] and snapshot taken at 200 ns point from the trajectory (opaque). The interacting residues are shown in licorice representation. This shows how the negatively charged interface in 1PRW is modified during the MD simulation. See main text for more details.
(TIF)

Figure S3Conformations sampled by 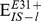 system. (a) RMSD of subunits depicted schematically in figure 1 (same color code) as well as the total structure (black) reveals that the N-lobe changes conformation, while the C-lobe and the linker are less mobile. The overall change in the protein conformation is relatively small, mainly following the change in the N-lobe perturbed by the protonation of E31. There is a single attempted jump at 155 ns. (b) That a single state near the initial conformation is sampled is clearly depicted by the (*φ*,*l*) plot.(TIF)
