# Supplementary figures and images for: Designing Molecular Dynamics Simulations to Shift Populations of the Conformational States of Calmodulin
Source: PLoS Comput Biol. 2013 Dec 5;9(12):e1003366. doi: 10.1371/journal.pcbi.1003366 (PMC3854495; doi:10.1371/journal.pcbi.1003366)

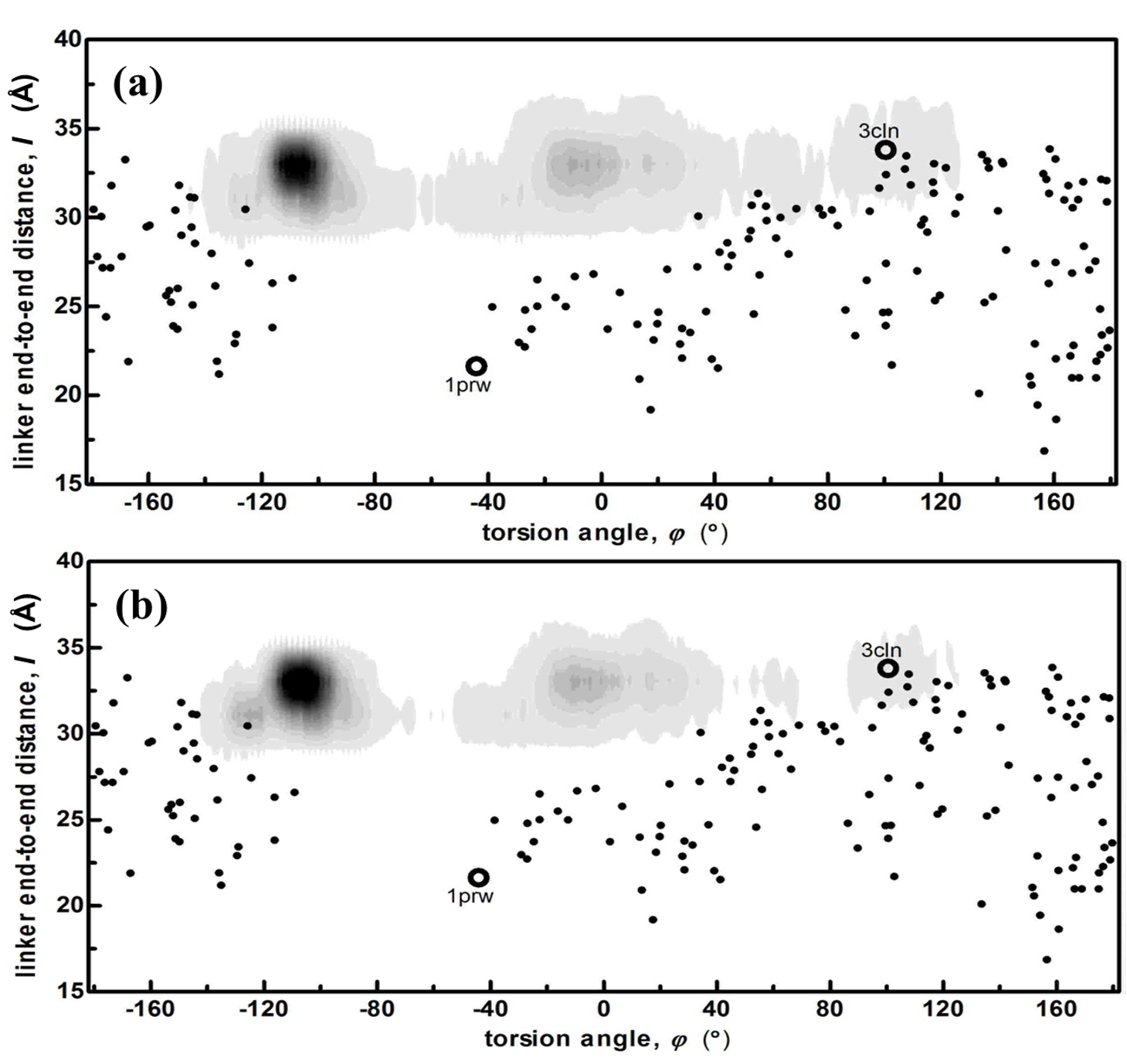

Supplement: Figure S1 — (φ,l) plots for the two runs mimicking the low IS/low pH environment, . (a) 200 ns run; (b) 100 ns run. Same sequence of events is observed in these systems. Their details are discussed in [24]. (TIF) [file pcbi.1003366.s001.tif]

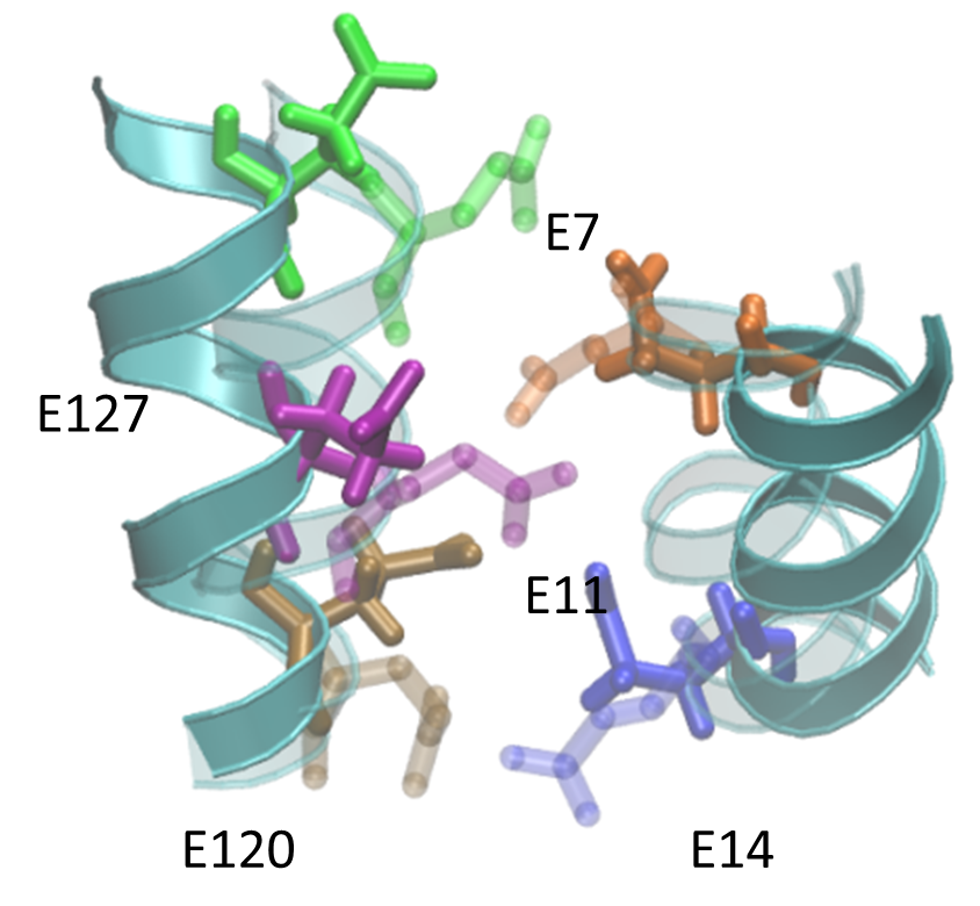

Supplement: Figure S2 — N- and C- lobe interface shown for the 1PRW crystal structure (transparent) [30] and snapshot taken at 200 ns point from the trajectory (opaque). The interacting residues are shown in licorice representation. This shows how the negatively charged interface in 1PRW is modified during the MD simulation. See main text for more details. (TIF) [file pcbi.1003366.s002.tif]

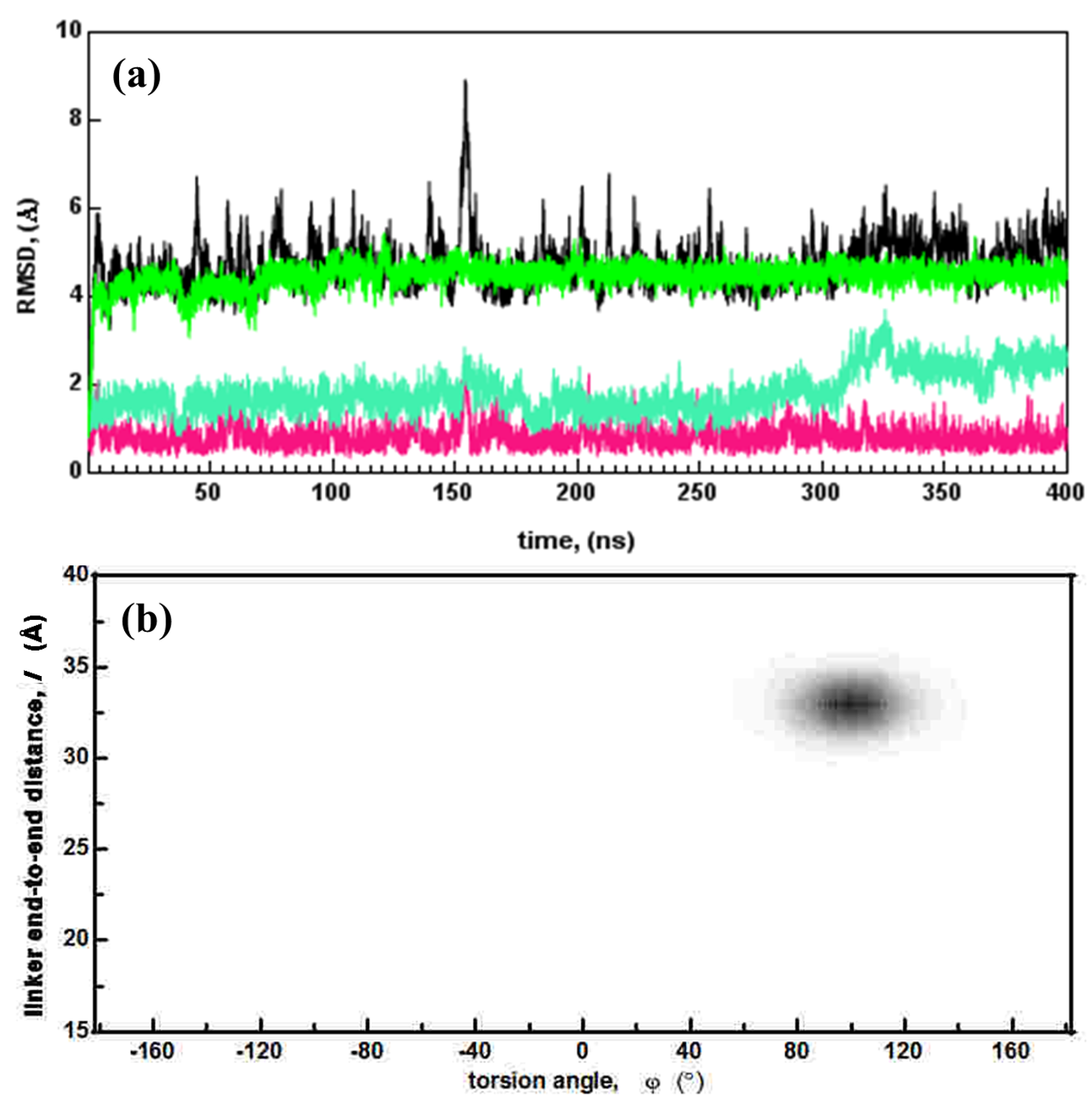

Supplement: Figure S3 — Conformations sampled by system. (a) RMSD of subunits depicted schematically in figure 1 (same color code) as well as the total structure (black) reveals that the N-lobe changes conformation, while the C-lobe and the linker are less mobile. The overall change in the protein conformation is relatively small, mainly following the change in the N-lobe perturbed by the protonation of E31. There is a single attempted jump at 155 ns. (b) That a single state near the initial conformation is sampled is clearly depicted by the (φ,l) plot. (TIF) [file pcbi.1003366.s003.tif]
